# Association of demographics, lumbar active range of motion and disability in chronic low back: a baseline data analysis of a randomized controlled trial from Pakistan

**DOI:** 10.1186/s12891-024-07613-9

**Published:** 2024-06-22

**Authors:** Aftab Ahmed Mirza Baig, Basit Ansari, Syed Imran Ahmed, Farhan Ishaque, Waqas Ahmed Farooqui

**Affiliations:** 1 Faculty of Health Sciences, Iqra University, North Campus, Karachi, Pakistan; 2 Department of Health, Physical Education and Sports Sciences, University of Karachi, Karachi, Pakistan; 3 Department of Physiotherapy, Dow Institute of Physical Medicine and Rehabilitation, Dow University of Health Sciences, Karachi, Pakistan; 4 School of Public Health, Dow University of Health Sciences, Karachi, Pakistan

**Keywords:** Backache, Disability evaluation, Lumbago, Movement, Physically disabled

## Abstract

**Background:**

The disability and significant economic costs accredited to Low back pain (LBP) are likely to rise which is an essential problem in low and middle-income countries like Pakistan. The associated factors of LBP are age, sex, and race including physical activity, high spinal load, lifting, bending, and twisting occupations. The literature highlighted there is substantial differences in associated factors of LBP within available studies in developing countries. The objective is to investigate the association of demographic factors and lumbar range of motion with disability in patients with chronic low back.

**Methods:**

A baseline data analysis was performed as an analytical cross-sectional study among 150 patients with chronic low back in a randomized controlled trial with a duration from March 2020 and January 2021. After recording demographics, Modified-Modified Schober’s test was used to measure lumbar flexion and extension and Oswestry disability index for disability. After the descriptive analysis the continuous variables, age and pain were analyzed with Spearman’s correlation. Variables that were significant in bivariate analysis were then fitted in a multivariable linear regression. The Kruskal–Wallis test was used to analyze variations of disability in gender, marital status, work status, education level, and duration of pain. The *p*-value of 0.05 was significant.

**Results:**

The results showed a significant correlation between age and pain in sitting (*rh*=-0.189, *p* = 0.021 and *rh* = 0.788, *p* < 0.001) with the disability but no significant effects of age and pain in sitting (*B*=-0.124, *p* = 0.212 and *B* = 1.128, *p* = 0.082) on disability were found. The decrease in lumbar flexion and extension was found to increase disability (*B*=-6.018 and − 4.032 respectively with *p* < 0.001). Female gender (x2(1) = 15.477, *p* = < 0.001) and unmarried marital status (x2(1) = 4.539, *p* = 0.033) had more disability than male gender and married marital status, respectively. There was a significance between groups of the duration of pain regarding disability (x2 (2) = 70.905, *p* < 0.001). Age, education level, and work status showed no significance (*p* > 0.05).

**Conclusions:**

The female gender and unmarried marital status are associated with functional disability. Decreased lumbar range of motion accompanies more disability, while age, education level, and work status do not effect on disability.

## Background

Chronic low back pain (CLBP) is a major cause of disability worldwide, and its impact is increasing as the population grows and ages [1]. It is defined by the location of pain, from the margins of lower rib to the creases of the buttock [1]. This is particularly problematic in low and middle-income countries like Pakistan, where resources and arrangements to deal with the issue are inadequate [1, 2].

It is estimated that around 60–80% of adults will experience low back pain (LBP) at some point in their lives. The global incidence rate of LBP is around 15% annually, with a point prevalence of 30% [3]. Approximately 5–10% of LBP cases will become chronic [4]. A report by the World Health Organization shows that 22% of patients experience chronic pain, with 48% of them being worried about their pain [4]. CLBP is often accompanied by disabilities in various demographics. The prevalence of CLBP increases as age progresses, with higher prevalence in women [4]. Non-specific causes of LBP can affect around 10–25% of young and middle-aged individuals [5]. Patients with CLBP have a 3% shorter life expectancy than healthy individuals to avoid pain [6]. Given that LBP is the most frequent reason for consultations in primary care, there is a strong case for increased efforts to improve healthcare for patients with this condition [7].

The disability and economic costs associated with LBP are expected to rise, making it an urgent issue that requires collaboration between people with LBP, policymakers, clinicians, and researchers. Together, they can work towards developing and implementing effective solutions to address the impact of disability and significant economic costs caused by LBP [8].

A review conducted on the topic of LBP identified various factors associated with this condition. These factors include age, sex, race, high intensity physical activity, high spinal load, lifting, bending, and twisting occupations. The review also highlighted that there are significant variations in the prevalence of LBP in developing countries [9]. Variations have been seen with LBP in occupation-related populations along with low frequency in individuals with a higher level of education [10]. Studies have shown that individuals in certain occupations such as nurses, cooks, drivers, school employees, office workers, and industrial employees are more vulnerable to LBP due to prolonged standing, heavy lifting, and lack of rest [11]. Recurring symptoms are common, with the majority of patients experiencing symptoms more than once a year [11]. However, there is a lack of research on the sociodemographic factors and lumbar range of motion associated with CLBP in developing countries like Pakistan. Therefore, this study aims to investigate the relationship between sociodemographic factors, lumbar range of motion, and disability in patients with CLBP in Pakistan.

## Methodology

### Subjects and study design

This study followed an analytical cross-sectional study design and involved the analysis of baseline data from a randomized controlled trial (RCT) [12] consisting of patients with CLBP. That RCT was conducted from March 2020 to January 2021, and received ethical approval from the Institutional Bioethical Committee (IBC) of Karachi University (KU), Karachi, Pakistan (IBC-KU-78/19). The RCT was prospectively registered on clinical.trial.Gov with ID: NCT04206137 (December 20, 2019). The researchers used purposive sampling to collect a representative sample of 150 patients with CLBP, aged 18 to 40 years, who had been experiencing pain for more than 3 months and had consulted with Sindh Institute of Physical Medicine and Rehabilitation, the former institute of Dow University of Health Sciences in Karachi, Pakistan. However, patients with certain red flags such as a history of spinal surgery, previous administration of epidural injections, LBP due to specific pathology, patients with neurological deficits (such as stroke), and those with any clinical disorder contraindicated to exercise, were excluded [5, 13]. Written consent was obtained from all the participants before they were enrolled in the study.

### Outcome assessment

The study consisted of various questions related to demographics (gender, age, marital status, education level, working status), including the location of pain, duration, and intensity on the Visual Analogue Scale (VAS-10 cm) [12]. Additionally, the functional disability was evaluated through the Oswestry Disability Index (ODI) questionnaire, and the range of motion of the lumbar region was measured using the Modified-Modified Schober’s test.

### Functional disability

The participants were asked to complete the ODI questionnaire in either English or Urdu to self-report any functional disability. This questionnaire is a reliable and standard tool to evaluate the effects of pain on daily activities. It provides a score ranging from 0 to 100, where an increase in score indicates increased disability. The cut-off value score of “9” has a sensitivity of 62% and a specificity of 55% [14].

### Flexion and extension range of motion

The range of motion for the trunk’s flexion and extension was assessed manually using a test known as the Modified-Modified Schober’s test. The assessor marked the posterior superior iliac spines (PSIS) on each patient using a body marker and marked a midline point (lower mark) between both PSIS. They then made an upper mark about 15 cm above the lower mark in the straight midline of the spine. To calculate lumbar flexion, the distance between these marks was measured while the patient was in a forward bending position, and then subtracted from the length measured while standing (15 cm). Similarly, to calculate lumbar extension, the same length between upper and lower marks was measured while the patient was in a backward bending position, and then subtracted from 15 cm [15].

### Statistical analysis

The data was analyzed using the Statistical Package for Social Sciences, version 21 (SPSS Inc., Chicago, Illinois, USA). Categorical variables were reported as frequency and percentages, while continuous variables were presented as means and standard deviation. The standard assumptions for data normal distribution were tested as not normal with Shapiro-Wilk test (*p* < 0.05). The frequency and percentages of reporting disability among study participants used a score of 0-<40% as mild to moderate disability, 40–60% as severe disability, and > 60% as very severe disability based on a previous study conducted among patients with CLBP [16]. The ODI (disability) score was analyzed as a continuous outcome variable and investigated for significant association with age and pain scores using Spearman’s correlation analysis. Variables that were significant in bivariate analysis were then fitted in a multivariable linear regression. The Kruskal–Wallis test was used to analyze variations of disability in gender, marital status, work status, education level, and duration of pain. The level of significance was 0.05.

### Power analysis for sample size

The power of test was calculated to justify the sample size of 150 samples using PASS version 2021 software, based on the multiple linear regression with 95% confidence of interval, 4 independent variables, 0.739 R2 computed from our study results. The power of the test was found to be more than 99%.

## Results

### Characteristics of participants

**Fig. 1:**
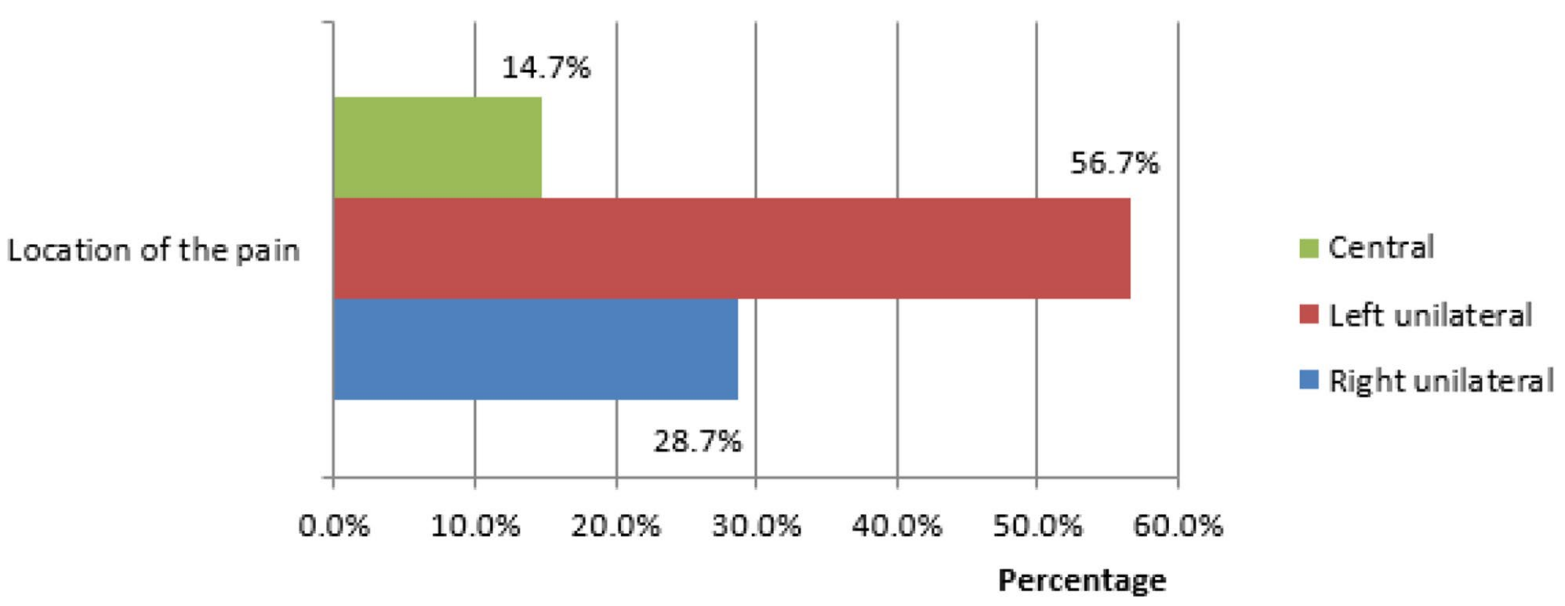
Types of Chronic low back pain according to location of pain

Among 150 patients most were males. Most of the participants had left-side unilateral CLBP than right-side. However the patients with central CLBP were less as shown in the figure (Fig. 1).

The mean age was 28.8 years. More than half (53.3%) were married and about two-thirds (80.7%) had a higher secondary education level or more. Most (60.7%) had a 3 to 12 months’ duration of pain (Table 1). The mean pain intensity in sitting was 3.65 ± 1.14 cm. The mean disability score was 33.97 ± 11.06. About 100(66.7%) of participants had mild to moderate disability and 50(33.4%) had severe disability. The mean lumbar flexion and extension measurements were 4.51 ± 0.93 cm and 2.27 ± 0.58 cm, respectively.

**Table 1:** General characteristics of the study participants

Variables	*N* = 150 (%)
Gender	
Male	90 (60)
Female	60 (40)
Age (years), Mean ± SD	28.8 ± 5.4
Marital status	
Married	80 (53.3)
Unmarried	70 (46.7)
Working status	
Sedentary work	27 (18)
Less sedentary work	36 (24)
Active work	87 (58)
Education level	
No education	6 (4)
Primary school education	7 (4.7)
Secondary school Education	16 (10.7)
Higher secondary school education	46 (30.7)
Graduation	42 (28)
Post-graduation	33 (22)
Duration of pain (months)	
3–12	91 (60.7)
>12–24	36 (24)
> 24	23 (15.3)

### Associations of age and pain in sitting with disability

The results in Table 2 showed a significant correlation between age and pain in sitting with the disability but according to the linear regression model, no significant effects of age and pain in sitting on disability were found. The lumbar flexion and extension both strongly negatively correlated with disability with significance in the linear regression model (Table 2).

**Table 2:** Spearman’s correlation and regression analysis of continuous variable with disability

Variables	Oswestry disability score for disability	
	*r*_s_ (*p*-value)	β (*p*-value)
**Age (years)**	-0.189 (0.021*)	-0.124(0.212)
**Pain intensity in sitting (VAS-cm)**	0.788 (< 0.001*)	1.128 (0.082)
**Lumbar flexion (cm)**	-0.832(< 0.001*)	-6.018(< 0.001*)
**Lumbar extension (cm)**	-0.688(< 0.001*)	-4.032 (< 0.001*)

Note: VAS = Visual Analogue Scale

### Association of female gender and unmarried marital status with disability

Table 3 clearly shows that females and unmarried individuals suffer from more disability (mean rank = 92.58 and 83.57 respectively) compared to males (mean rank = 64.12) and married individuals (mean rank = 68.44), respectively. These differences are statistically significant, as indicated by the Kruskal-Wallis H test.

**Table 3:** Oswestry disability score variations by demographic and clinical characteristics of patients with CLBP

Variables	ODI Scores (Mean ± SD)	${\varvec{\chi }}^{2}$(df)	*P* value	
**Gender**				
Female	38.18 ± 10.2	15.477 (1)	< 0.001*	
Male	31.16 ± 10.7			
**Marital status**				
Unmarried	36.18 ± 11.3	4.539 (1)	0.033*	
Married	32.02 ± 10.5			
**Work status**				
Mostly sedentary	31.75 ± 11.2	1.213 (2)	0.545	
Sedentary	33.87 ± 10.3			
Active	34.69 ± 11.4			
**Education level**				
No education	35.66 ± 10.3	1.068 (5)	0.957	
Primary	31.80 ± 12.6			
Secondary	32.76 ± 11.9			
Higher Secondary	33.57 ± 12.6			
Graduation	33.98 ± 10.4			
Post-graduation	35.23 ± 9.5			
**Duration of pain (months)**				
3–12	40.08 ± 8.4	70.905 (2)	< 0.001*	
>12–24	24.52 ± 7.6			
>24	24.54 ± 7.5			

### Association of duration of pain with disability

Table 3 displays a marked contrast in disability scores among the groups categorized by pain duration (χ2 (2) = 70.905, *p* < 0.001). Specifically, the average rank disability score was 99.53 for the 3 to 12-month pain group, 38.56 for the more than 12 to 24 months’ pain group, and 38.26 for the more than 24 months’ group.

## Discussion

The current study determined the relationship of demographical factors and lumbar flexion and extension with CLBP disability among patients with CLBP. This rehabilitation center-based study found more than 60% of patients with CLBP had mild to moderate disability and one third of patients had severe disability. Factors independently associated with disability in the current study of CLBP patients were gender, marital status, duration of pain, and lumbar flexion and extension.

The mean ODI score in the current study was 33.97 which is in a category of mild to moderate disability suggesting patients with more disability with functional activities [16, 17]. This category suggests personal care, sleeping, and sexual activity are not grossly affected [18]. Six out of ten participants had mild to moderate disability levels and almost three out of ten had severe disability. The patients with CLBP in Karachi therefore, have increased disability and are consistent with findings in other settings [16, 19, 20]. The results of the current study support the probable applicability and validity of the ODI score in the Pakistani context.

It is recognized that males and females have dissimilar behavioral and physiological responses to pain. Females are more prone to CLBP showing the worst response leading to disability [21]. The current study observed that more disability accounted for females amongst enrolled patients. In Cameroon, a cross-sectional study of patients with LBP of at least 12 weeks in a tertiary hospital reported a negative association with males [21], thus in line with the finding of the current study. In Spain, the female gender is also highly frequent reporting CLBP leading to disability [22].

The relationship between marital status and disability appears ambiguous. A cross-sectional study as a part of a three and half-year cohort study among patients with CLBP found that marital status as living with a partner is less disable to do personal activities as compare to living alone. The participants of that study were older-aged as compare to the current study [23]. Similar to the findings of the current study, the tertiary hospital-based study found married (as living with a partner) having more disability as compared to unmarried (living alone) among patients with CLBP [21]. It might be due to the more demanding working schedule of married individuals to overcome responsibilities of spouse and children.

However, the variability in disability score in current patients was more influenced by duration of pain and lumbar range of motion. In a Saudi Arabian study on multi-dimensional profiles for patients with CLBP, the increased pain intensity was found to increase disability [24]. Similarly, in the Republic of Korean office workers with CLBP, a low to moderate correlation of pain with the ODI scores have been shown [25]. The pain intensity in sitting significantly correlated but did not contributed to disability in patients enrolled in the current study. This change in finding might be due to the pain intensity as positional with sitting in the current study.

The duration of LBP has been suggested to affect the disability. Evidence from one study found that patients with LBP having more duration of pain were more likely to suffer higher levels of disability [22]. The findings of the current study confirm this and demonstrate that duration of pain even in chronicity has a significant effect on disability.

The lumbar ROM is known to influence disability and can lead to chronicity of LBP. A cross-sectional study found LBP disability as predicted by the decrease in the overall lumbar range of motion with significance [26]. Cross-sectional, an ancillary study of an international multicenter epidemiological study found flexion as a less significant influencing factor for disability [27] and another cross-section study found a significant correlation of extension with disability [28]. However, the results of the current study found that the decrease in both flexion and extension ROM increases disability but there was more influence of flexion ROM than extension.

Age has also been documented to influence disability in LBP [16, 19]. The current study observed a weak negative correlation between age and disability only in bivariate analysis. Furthermore, contradictory to this age has been implicated in increased pain related-disability [20, 21]. The difference in the results might be due to the different and small range of age groups.

Educational level has been related to LBP disability. Lower educational level has been found as a demographic factor related to disability due to CLBP [22]. Furthermore, a longitudinal field study has found education as a predictor for disability in CLBP [29]. Despite findings with previous studies [20, 21, 29], the current study found no association between level of education and disability.

The working status with physical work demands has also been associated with disability due to LBP [30]. The name of this variable varies in previous studies with categories [19, 21, 24].

The study from the Nigerian hospital found more disability among employed than no employed with a significant correlation [19]. However in line with current study findings the Cameroon and Saudi Arabian studies found a non-significant association between disability and work status [21, 24].

### Study limitations

There are many limitations in the current study. Important to report is study design as cross-sectional restricted the formation of causal relationships, this could be suitable with a prospective cohort design. However, the current study discovered associations that can aid as the point of reference for future studies. Another limitation is the possibility of selection bias due to the non-probability purposive sampling and rehabilitation center-based nature of the study. Therefore, it is probable that the findings of the current study might not direct the characteristics of patients with CLBP at other health care settings throughout the county. Hence, the current study findings must be generalized with careful attention and interpretation.

Though, the study used demanding statistics to investigate the association of sociodemographic factors and lumbar range of motion with disability in patients with CLBP. The current study to the best of the author’s knowledge is the first in Pakistan with this aim and so could aid as the basis for research ahead.

## Conclusion

The evidence from this study has confirmed that CLBP disability is associated with female gender and unmarried marital status. Decreased lumbar flexion and extension range of motion associates with more disability, while age, education level and work status had no effect on change in disability. The results of current study suggest a context-specific indication for priority setting in prevention and treatment plans to decrease the CLBP burden. The larger sample, population-based studies are necessary to modify current results.

## Data Availability

The data is available from corresponding author on reasonable request.

## Abbreviations

LBP: Low Back Pain
CLBP: Chronic Low Back Pain
ROM: Range of Motion
IBC: Institutional Bioethical Committee
KU: Karachi University
ODI: Oswestry disability index
PSIS: Posterior Superior Iliac Spines
